# SARS-CoV-2 specific plasma cells acquire long-lived phenotypes in human bone marrow

**DOI:** 10.1016/j.ebiom.2023.104735

**Published:** 2023-08-08

**Authors:** Axel R. Schulz, Leonard Fiebig, Heike Hirseland, Lisa-Marie Diekmann, Simon Reinke, Sebastian Hardt, Antonia Niedobitek, Henrik E. Mei

**Affiliations:** a Deutsches Rheuma-Forschungszentrum Berlin, A Leibniz Institute, Berlin, Germany; b Cell Harvesting Core, Berlin Institute of Health, Berlin, Germany; c Center for Musculoskeletal Surgery, Charité Universitätsmedizin Berlin, Berlin, Germany

**Keywords:** SARS-CoV-2, Human bone marrow, Long-lived plasma cell, Humoral memory, CD19, CD45

## Abstract

**Background:**

SARS-CoV-2 specific antibody-secreting plasma cells (PC) mediating specific humoral immunity have been identified in the human bone marrow (BM) after COVID-19 or vaccination against SARS-CoV-2. However, it remained unclear whether or not they acquire phenotypes of human memory plasma cells.

**Methods:**

SARS-CoV-2-specific human bone marrow plasma cells (BMPC) were characterised by tetramer-based, antigen-specific flow cytometry and FluoroSpot assay.

**Findings:**

SARS-CoV-2 spike-S1-specific PC were detectable in all tested BM samples of previously vaccinated individuals, representing 0.22% of total BMPC. The majority of SARS-CoV-2-specific BMPC expressed IgG and their specificity for the spike S1 protein indicated emergence from a systemic vaccination response. Of note, one-fifth of SARS-CoV-2-specific BMPC showed the phenotype of memory plasma cells, i.e., downregulated CD19 and present or absent CD45 expression.

**Interpretation:**

Our data indicate the establishment of phenotypically diverse SARS-CoV-2-specific PC in the human BM after basic mRNA immunization, including the formation of memory phenotypes. These results suggest the induction of durable humoral immunity after basic mRNA vaccination against SARS-CoV-2.

**Funding:**

The study was supported by funding by the 10.13039/501100001659DFG grants TRR130 TP24, ME 3644/8-1, and the Berlin Senate. SR received funding from 10.13039/501100001659DFGSFB-1444 C01 Central Service Project.

**Research in context**

**Evidence before this study**

SARS-CoV-2 specific antibody-secreting plasma cells have been described in the human bone marrow after COVID-19 infection and vaccination. We previously described distinct phenotypes of human bone marrow plasma cells with stable versus dynamic properties.

**Added value of this study**

20% of vaccination-induced SARS-CoV-2-specific BM plasma cells acquire CD19^−^CD45^+/−^ phenotypes associated with durability.

**Implications of all the available evidence**

Basic mRNA vaccination with 3–4 doses are sufficient to induce memory plasma cells in man. Antigen-specific humoral immunity is based on an array of plasma cells.

## Introduction

Long-term serological immunity relies on persistent antigen-specific antibody-secreting plasma cells (PC) and memory B cells, the latter of which can give rise to large numbers of additional plasma cells after re-stimulation by cognate antigen.^1^ While the emergence of specific memory B cells can be analysed in humans by subsets circulating in the blood,^2^, ^3^, ^4^ memory PC do not recirculate, and persist as resting, continuously antibody-secreting cells in the BM for extended periods of time, perhaps for a lifetime.^1^^,^^5^^,^^6^

We and others previously characterised human PC lacking the expression of the B cell surface antigen CD19,^4^^,^^7^, ^8^, ^9^ altogether indicating that PC lacking CD19 are, or comprise, long-lived PC responsible for the observed stability of specific serum antibody (Ab) titres.^10^ In support of exceptional durability of individual CD19-negative PC, radiocarbon dating of human mucosal lamina propria PC suggested CD19^−^CD45^+^ PC to be at average eleven, and CD19^−^CD45^−^ PC to be twenty-two years old, while CD19^+^ PC showed contemporary levels of radiocarbon.^9^ Different from CD19^+^ PC, PC lacking CD19 are enriched in the human bone marrow, mainly secrete IgG relevant for serological immunity, comprise few if any proliferating cells, exhibit a pro-survival CD95^−^/Bcl-2^high^ phenotype, and secrete antibodies specific for childhood vaccines.^4^^,^^7^ Recent reports of BMPC specific for SARS-CoV-2 indicate successful homing to the human BM after SARS-CoV-2 challenge by infection or vaccination.^11^^,^^12^ In addition, the durability of SARS-CoV-2-specific antibodies in the blood serum exceeds the 3-week-half-life of IgG molecules,^13^^,^^14^ implying the continued presence of PC replenishing serum Ab titres after the acute immune response has waned.

## Methods

### Bone marrow samples

Native human bone marrow (BM) was obtained during hip joint replacement surgery from 22 patients, as described before.^4^ Information on the donors is summarised in Supplementary Table S1. Sex was self-reported by study participants.

In 17 cases, paired blood samples were collected prior to surgery in EDTA-coated tubes.

### Single-cell suspension from human bone marrow samples

Single-cell suspensions were isolated in a safety class S2 workbench. First, larger fragments of spongious bone were chopped with a scalpel in a sterile Petri dish (Corning). All material was then collected in a 50 mL centrifuge tube (Falcon, BD Biosciences).

PBE (PBS supplemented with 0.2% BSA and 5 mM EDTA) was added to the bone marrow material to liberate cells from bone. After gentle agitation by inverting the tube, the suspension was filtered through a 70 μm cell strainer (BD Biosciences), into a new 50 mL centrifuge tube, to remove cell clumps, fat, and solid bone fragments. Larger bone fragments were processed again to recover additional cells.

Afterwards, the cell suspension was centrifuged at 4 °C, 300×*g*, for 10 min. The supernatant was removed by vacuum aspiration. The cell pellet was resuspended in 30 mL PBE.

Mononuclear BM cells were isolated by density gradient centrifugation, by carefully overlaying the filtered BM cell suspension onto 15 mL Ficoll-Paque-Plus (GE healthcare, density, 1.077 g/mL) at room temperature, followed by centrifugation for 20 min at room temperature without brake and low acceleration, at 800×*g*. Then, the interphase was collected using a 25 mL serological pipette (Corning) and transferred into a new 50 mL centrifuge tube. After filling up to 50 mL with PBE, the cells were centrifuged at 4 °C, 300×*g*, for 10 min. The cell pellet was resuspended in 10 mL PBE, and cells were counted volumetrically using a MACSQuant 10 (Miltenyi Biotech). Then, aliquots of 5 × 10^6^ cells were prepared in 1 mL cryostorage tubes (LVL technologies), pelleted (300×*g*, 4 °C, 10 min) and resuspended in 400 μL fetal calf serum (FCS, Corning). Then, 560 μL proteomic stabilizer (Smart Tube Inc., Las Vegas, CA) were added and immediately mixed in by pipetting, facilitating fixation of the cells. After incubation for 12 min at room temperature, tubes were cryopreserved at −80 °C.

### PBMC isolation from blood

PBMC were isolated from whole blood by density gradient centrifugation as previously described,^4^ and further processed as described for bone marrow cell suspensions.

### Flow cytometry

Antigen-loaded tetramers were prepared as outlined in Supplementary Table S2 by incubating biotinylated SARS-CoV-2 Spike S1 (aa Val16-Arg685, Accession #QHD43416.1, Biolegend), receptor binding domain (RBD) (aa Arg319-Phe541, Accession# QHD43416.1, Biolegend), or nucleocapsid (NP) (aa Ser2–Ala419, Accession# WGJ79622.1, Miltenyi Biotech) with respective fluorochrome-tagged streptavidins (Biolegend) at a molecular ratio of 4:1 for at least 2 h at 4 °C in the dark. Prior to mixing with the intracellular staining cocktail, tetramer solutions were incubated 1:2 with 40 μM D-Biotin (Thermo Fisher, pre-diluted in PBS) for 5 min at room temperature to saturate unbound streptavidin.

Antibodies used in this study (Supplementary Table S2) were all from commercial sources. Optimal antibody staining concentrations were determined by antibody titration and antibody single stainings were used to assess signal spillover.

Fixed and cryopreserved BM mononuclear cells (BMMC) were thawed for 5 min in cold water (10–15 °C), washed twice in 4 mL PBA buffer (PBS supplemented with 0.5% BSA and 0.02% sodium azide), pelleted at 700×*g*, for 7 min, at 4 °C, resuspended in 2 mL PBA, and counted volumetrically on a MACSQuant 16 (Miltenyi Biotech). Up to 15 × 10^6^ cells were transferred to a FACS-Tube (Sarstedt), pelleted and resuspended in 80 μL PBA supplemented with 10 units heparin (Ratiopharm, Ulm, Germany). Next, cells were incubated for 15 min at room temperature, followed by a 30 min incubation at room temperature in the dark with 100 μL surface staining cocktail (Supplementary Table S2). Afterwards, cells were washed once with up to 4 mL PBA (700×*g*, 7 min, 4 °C) and resuspended in 2 mL 1X permeabilization buffer (prepared 1:10 from 10X eBioscience Permeabilization Buffer (Thermo Fisher Scientific) diluted in millipore water). Subsequently, cells were pelleted at 700×*g*, for 10 min, at 4 °C, and resuspended in 200 μL permeabilization buffer supplemented with the intracellular staining cocktail (Supplementary Table S2) and 10 units heparin, followed by a 30 min incubation at room temperature in the dark. Next, cells were washed once with 4 mL permeabilization buffer and once with 4 mL PBA. Finally, 380 μL of the cell suspension were acquired on a MACSQuant 16. Fixed and cryopreserved PBMC were processed in the same manner as BMMC, except for the addition of Fc-block (Miltenyi Biotech) during heparin incubation and antibody stainings. Antibody cocktails for PBMC stainings are outlined in Supplementary Table S2.

### FluoroSpot assay

The FluoroSpot assay was performed to confirm the presence of functional antibody-secreting cells specific for SARS-CoV-2 Spike S1 antigen. Single-cell suspensions from human BM samples were counted, and stained with a cell-surface antibody panel (Supplementary Table S2) for 20 min at 4 °C, followed by a washing step with PBE (300×*g*, 10 min, 4 °C). Next, BMMC were filtered through a 20 μm cell strainer (Miltenyi Biotech), adjusted to a concentration of 1 × 10^7^ cells/mL in PBE, supplemented with 1:1000 propidium iodide (PI) (Sigma, 1 mg/mL), and loaded into a primed MACSQuant Tyto Cartridge HS (Miltenyi Biotech). Next, live CD3^−^CD14^−^CD10^−^CD235a^−^PI^−^CD38^++^CD319^+^ PC were sorted from BMMC at 12 °C using a MACSQuant Tyto Cell Sorter (Miltenyi Biotech). The highly enriched PC fraction was collected from the sorting cartridge, adjusted to a total volume of 1 mL with PBE, counted volumetrically using a MACSQuant 10, and centrifuged 300×*g*, 10 min at 4 °C. After aspiration of the supernatant, cell pellets were resuspended in media (RPMI 1640 (1X) supplemented with GlutaMAX™–I (Gibco), 10% FCS (Corning), 100 U/mL penicillin-streptomycin (Gibco)) at cell concentrations ranging between 2000 cells/100 μL and 73,500 cells/100 μL and distributed to a previously prepared 96-well FluoroSpot plate. FluoroSpot plates were prepared as follows: A low fluorescent PVDF FluoroSpot plate (Mabtech) was treated with 15 μL 35% ethanol/well (diluted with water from 99.8% ethanol, Roth) for max. 1 min. 200 μL purified water (Milli-Q® IQ 7000 system, Merck) was added, and the plate was washed four times with purified water and coated with 100 μL/well as follows: For the BSA control, BSA (MACS® BSA stock solution, Miltenyi Biotech) was diluted to 15 μg/mL in PBS. As a technical positive control a mixture of anti-human IgA (MT57, Mabtech), anti-human IgG (MT91/145, Mabtech) and anti-human IgM (MT11/12, Mabtech) was used, and for coating each antibody was diluted to 15 μg/mL in PBS in the same tube. For detection of Spike-S1-specific BMPC, biotinylated recombinant SARS-CoV-2 protein S1 (carrier-free) (0.55 mg/mL, Biolegend) was diluted to 10 μg/mL in PBS. For coating, the plate was incubated at 4–8 °C overnight. The solution was removed from the wells by gently tapping the plate dry on a clean absorbent towel and washed five times with 200 μL/well PBS. Next, the plate was conditioned with 200 μL/well sterile RPMI for at least 30 min at RT. The medium was removed and 100 μL/well cell suspension was transferred to the plate. The plate was incubated at 37 °C, 5% CO_2_ for 16–24 h in an incubator. After that, the cell suspension was removed and the wells were washed five times with 200 μL/well PBS. For detection, anti-human IgA-490 (MT20, FITC, Mabtech), anti-human IgG-550 (MT78/145, Cy3, Mabtech) and anti-human IgM-640 (MT22, Cy5, Mabtech) were diluted 1:500 in PBS+0.5% FCS in one tube. Next, 100 μL of the detection Ab mix was transferred to each well and the plate was incubated for 2 h in the dark at RT. The solution was removed and the plate was washed five times with 200 μL/well PBS. After removing the PBS, 50 μL of fluorescence enhancer-II (Mabtech) was transferred to each well and the plate was incubated for 15 min in the dark at RT. The fluorescence enhancer-II was removed by flicking and tapping the plate. Last, the soft plastic from the bottom of the plate was removed and the plate was dried overnight in the dark at RT. FluoroSpot plates were analysed using an EliSpot/FluoroSpot Reader (AID).

### Data analysis and statistics

Flow cytometry data were stored in FCS3.1 files and analysed using FlowJo Version 10 (BD, TreeStar, Ashland, OR) and OMIQ.ai (Santa Clara, CA, USA) by manual gating as shown in Supplementary Figure S1. Prism version 9 (GraphPad software, San Diego, CA) was used to display data, and perform descriptive statistics and significance testing. FluoroSpot plates were analysed using the AID EliSpot reader 7.0 (AID) software.

### Ethics

All participants provided written informed consent to participate in this research. The studies were approved by the ethics committee of the Charité Universitätsmedizin Berlin (application number: EA2/123/21). All research was performed in alignment with the principles of the Helsinki Declaration.

### Statistics

Statistical tests and multiple testing correction procedures are stated in the figure legends. Exclusion criteria for donors/samples were a previous or current leukemia diagnosis, chemotherapeutic treatments or treatment with B-cell depleting therapies. Three out of 20 BM samples were excluded due to unexpected distributions of kappa versus lambda light chain expressing PC. One sample was excluded because it was retrospectively identified to have been obtained during the acute immune response 7 days after mRNA vaccination. Frequencies of reported PC subsets did not significantly differ between the donor groups stratified by sex.

### Role of funders

The funders did not have any role in the study design, data collection, data analyses, interpretation, or writing of this article.

## Results

**Fig. 1:**
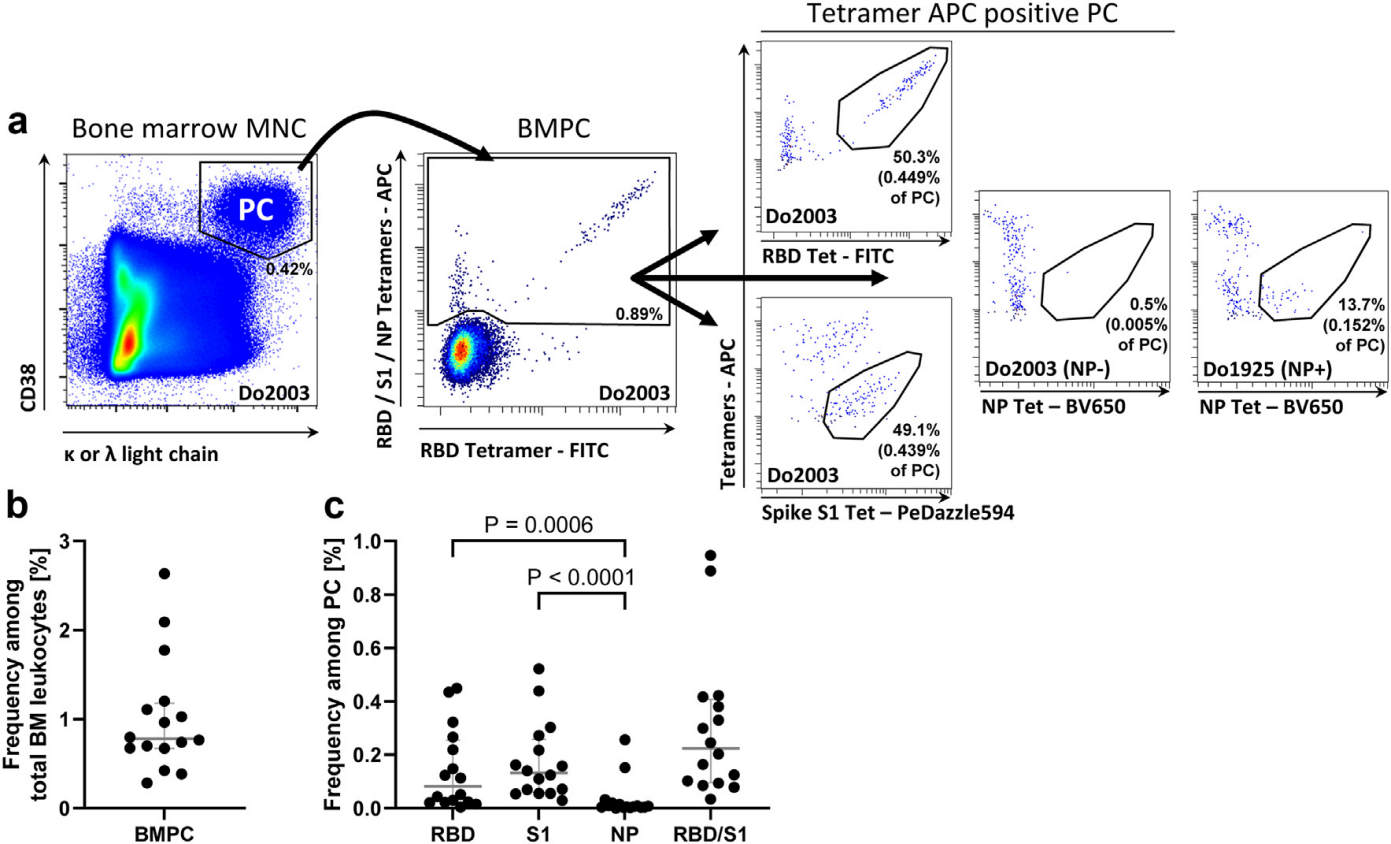
**Detection of SARS-CoV-2-specific bone marrow plasma cells.** (a) CD38^++^κ/λ light chain^++^ BMPC were gated as shown (extended in Supplementary Figure S1) and analysed for specificity against RBD, spike S1 (non-RBD domain), and NP by combinatorial tetramer staining. Data is from donor 2003 with undetectable NP-specific PC. Data of donor 1925 with evidence of NP-specific PC is shown as control. (b) Frequency of PC in total BM cells. (c) Frequencies of RBD-, S1- and NP-specific and RBD/S1-specific cells among BMPC. Frequencies of RBD/S1-specific PC correspond to the sum of RBD- and spike S1 (non-RBD domain)-specific PC. Medians and IQR are indicated. Friedman test with Dunn's multiple test correction was applied to compare the frequencies of RBD-, S1-, and NP-specific PC. Data of 16 donors are shown.

We here assessed the emergence of SARS-CoV-2 specific PC in the human BM in a total of 20 femoral head samples (11 women and 9 men, average age 70 years, range 50–87 years) obtained in March–November 2022, by flow cytometry (Supplementary Table S1). Three samples were excluded from analysis due to aberrant distributions of kappa versus lambda light chain expressing PC. One sample was excluded because it was obtained during the acute immune response 7 days after mRNA vaccination. Of the remaining donors, 14 reported having received three or four vaccinations, mostly with mRNA vaccines, against COVID-19. Two donors did not disclose vaccination information. Three donors reported prior infection with SARS-CoV-2 in addition to vaccinations. BMPC were detected according to high expression of CD38, and co-expression of antibody kappa or lambda antibody light chains, as described before (Fig. 1A, Supplementary S1).^15^ High forward scatter signals and expression of CD27 confirmed PC identity (Supplementary Figure S1). BMPC accounted for a median of 0.78% of BM mononuclear cells, and data of approximately 10.700–84.500 plasma cells were available for the analyses from individual donors (Fig. 1B, Supplementary Table S1). Spike S1-, RBD- or NP-specific PC were revealed by dual tetramer staining as established before and shown in Fig. 1A and Supplementary Figure S2.^16^ Consistent with previous reports employing EliSpot assays,^11^^,^^12^ SARS-CoV-2 spike-S1-specific PC (RBD/S1) were detectable at frequencies of 0.22% (median, range 0.03–0.94%) (Fig. 1C). Of these spike-S1-specific PC, 39% were specific for RBD (corresponding to a median of 0.08% of all BMPC) and 61% for the non-RBD-domain of S1 (corresponding to a median of 0.13% of all BMPC), indicating the selection of cells raised against immunodominant and potentially neutralizing SARS-CoV-2 epitopes into the pool of BMPC. We confirmed the presence of SARS-CoV-2 spike S1-specific BMPC in two additional donors by functional FluoroSpot assay, with concordant results compared with flow cytometric detection (Supplementary Figure S3). Substantial frequencies of more than 0.01% of nucleocapsid-specific PC were detected in 6 out of 16 individuals and at comparably low frequencies ((median 0.03%, maximum 0.26% for the 6 donors with evidence of NP-specific BMPC), Fig. 1C, Supplementary Table S1), suggesting a subordinate dominance of NP-specific PC compared to S1/RBD-specific PC in those 6 donors. Because of the limited number of NP-positive donors and the low frequency of NP-specific PC, subsequent analyses focused on RBD/S1-specific PC.

**Fig. 2:**
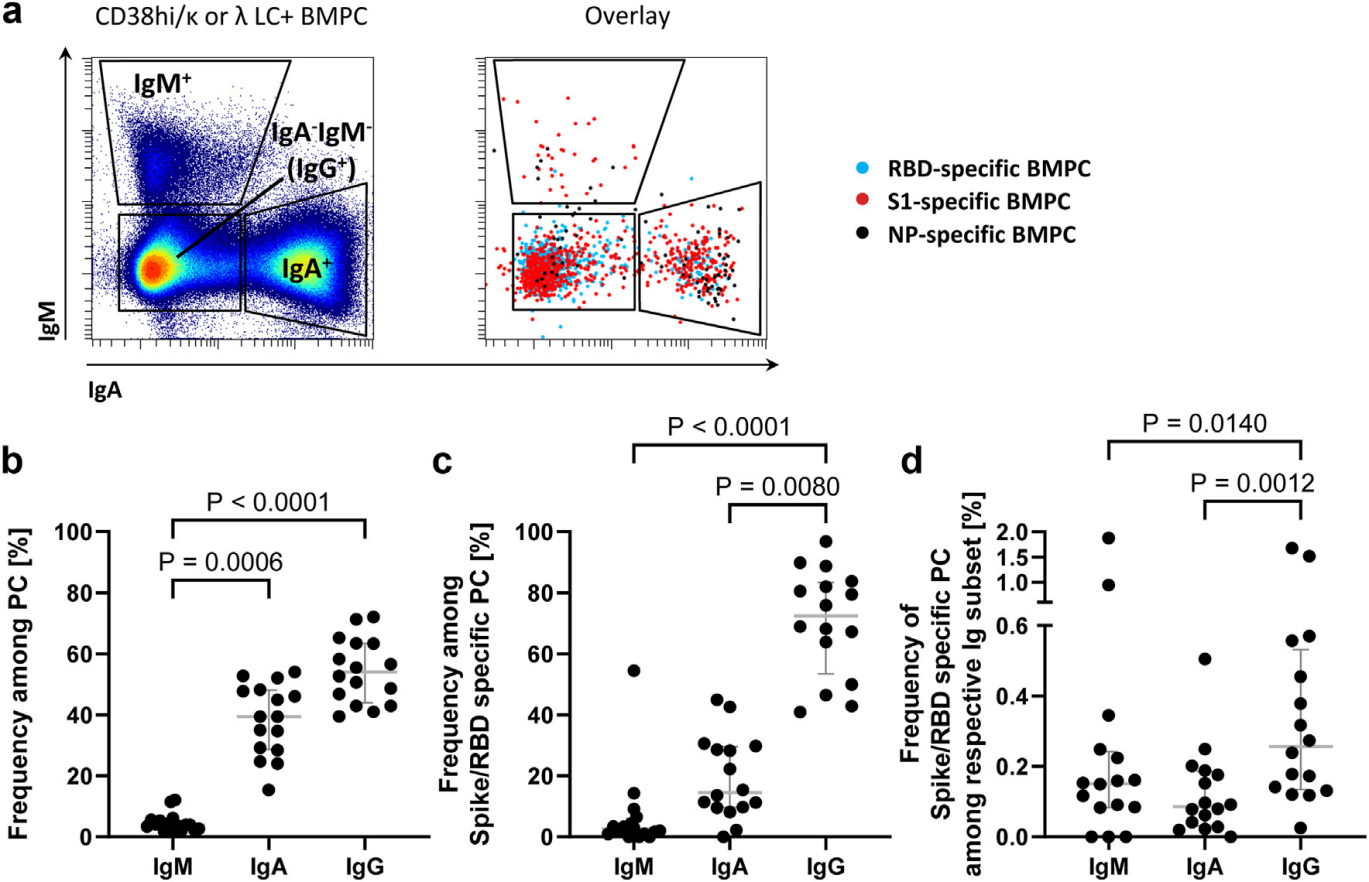
**Most SARS-CoV-2 spike S1-specific BMPC express IgG.** (a) Expression of IgA and IgM by total and SARS-CoV-2 specific BMPC. IgA^−^IgM^−^ BMPC are considered IgG^+^ PC, based on previous data and scarcity of IgE^+^ or IgD^+^ PC (<3% of BMPC,^4^^,^^17^ and unpublished data). Dot plots display concatenated data of all donors. Summary for total (b) and RBD/S1 BMPC (c). (d) Frequencies of RBD/S1 BMPC among BMPC expressing different Ig isotypes. Medians and IQR are indicated. Friedman test with Dunn's multiple test correction was applied. Data of 16 donors are shown.

In support of RBD/S1-specific BMPC emerging from systemic recall responses, most expressed IgG (median 72%) (defined by the absence of IgA and IgM expression), and IgG^+^ BMPC were enriched for RBD/S1-specific PC. 15% of RBD/S1-specific BMPC expressed IgA and only a few IgM (median 3%) (Fig. 2A–D).

**Fig. 3:**
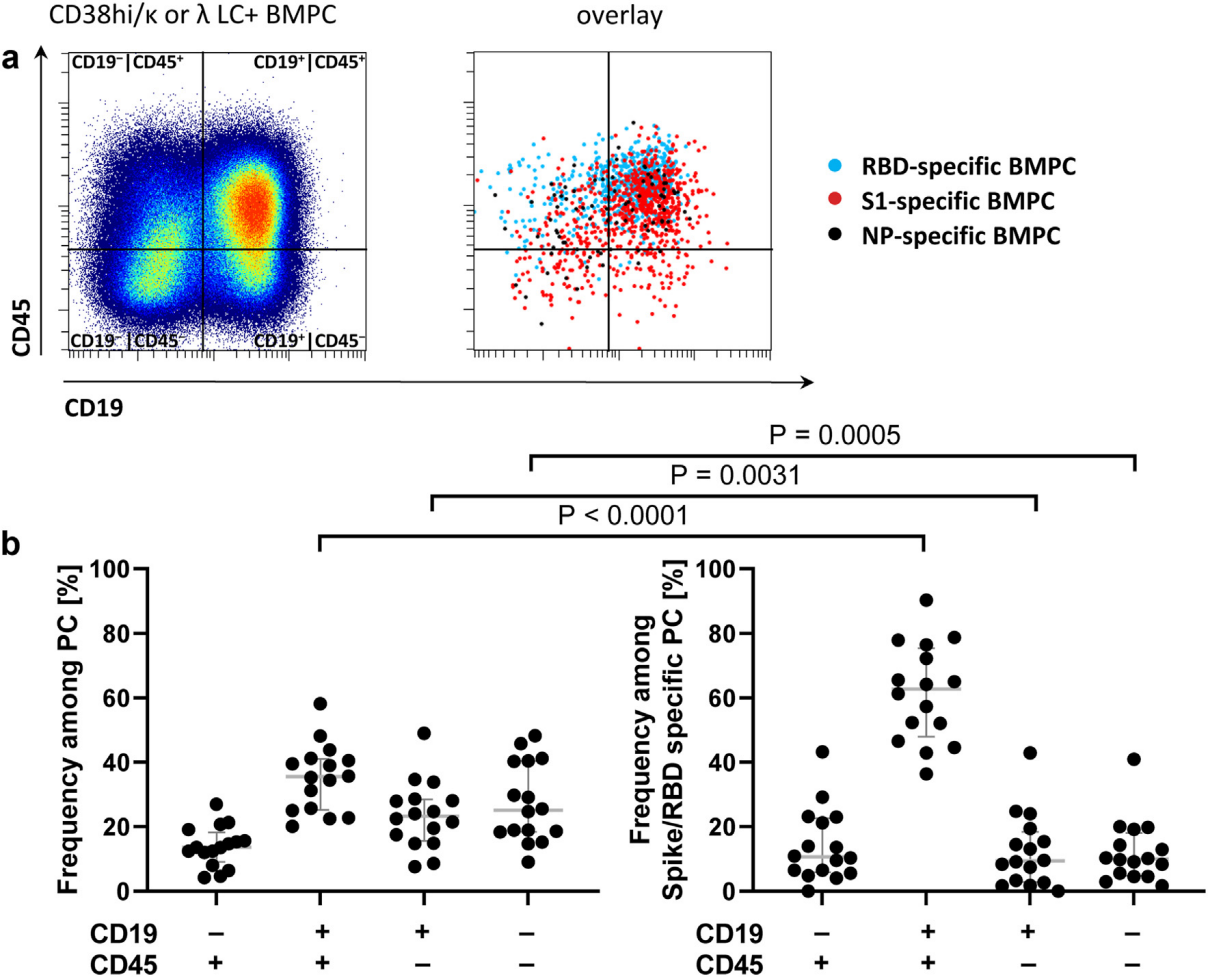
**SARS-CoV-2 spike S1-specific BMPC differentially express CD19 and CD45.** (a) Expression of CD19 and CD45 by total and SARS-CoV-2-specific BMPC. Dot plots display concatenated data of all donors. (b) CD19 and CD45 expression of total and RBD/S1-specific BMPC. Medians and IQR are indicated. Mann–Whitney testing was applied. Data of 16 donors are shown.

Total BMPC showed differential expression of CD19 and CD45, confirming previous reports of mature PC^4^^,^^7^^,^^9^ with a median of 36% expressing the conventional CD19^+^CD45^+^ phenotype, next to CD19^+^CD45^−^ (23%), CD19^−^CD45^−^ (25%), and CD19^−^CD45^+^ (16%) PC phenotypes, the latter two types being indicative of advanced PC maturation and longevity (Fig. 3A and B).^4^^,^^9^ Significant fractions of RBD/S1-specific PC also expressed these non-canonical PC phenotypes, comprising CD19^−^CD45^+^ (median 11%), and CD19^−^CD45^−^ (10%), and thus share the previously described phenotype of long-lived PC (Fig. 3B, Supplementary Figure S2). In line with limited antigen experience after 3–4 doses of vaccination, SARS-CoV-2-specific PC comprised lower frequencies of the non-canonical, longevity-associated phenotypes compared to overall BMPC (Fig. 3B).

In paired blood samples available from 13 of the 16 included donors, plasmablasts were detectable at low levels (median frequency, 1.9% of CD19^+^ B cells, Supplementary Table S1), comparable to amounts observed during steady-state^15^ indicating the absence of immune activation at the time of BMPC sampling that may have confounded the analyses. In further support of the absence of an acute SARS-CoV-2-specific immune response in our donors, only eight spike-S1-specific plasmablasts were found in the blood of a total of 4 out of 13 individuals, and none in the other 9 individuals, while class-switched memory B cells comprised a median of 0.24% spike-S1-specific cells (Supplementary Table S1). In addition, only HLA-DR^−^ BMPC were considered in our analysis. Together, these data argue against the possibility that SARS-CoV-2-specific PC observed in the BM samples were infiltrating plasmablasts from the blood, and support the presence of phenotypically diverse SARS-CoV-2 specific BM-resident PC.

## Discussion

We here show for the first time that basic mRNA vaccination against COVID-19 elicits a phenotypically diverse population of SARS-CoV-2-specific PC in the human bone marrow. SARS-CoV-2-specific BMPC comprise a 20% population of PC lacking CD19 with or without expression of CD45, that is, phenotypes previously linked to PC population stability, increased survival potential and longevity.

Obviously, SARS-CoV-2-specific CD19^+^CD45^+^ PC phenotypes were also identified and thus contribute to specific serological protection. Their bare presence in the bone marrow suggests that also these CD19^+^CD45^+^ PC have survived considerable time under steady-state conditions, in the absence of acute plasmablast bursts, as previously discussed.^4^^,^^18^ However, previous studies demonstrated total numbers of CD19^+^ BMPC to be more dynamic, likely reflecting the adaptability of the CD19^+^ plasma cell compartment to emerging challenges.^4^ Unlike CD19^−^ PC, CD19^+^ BMPC were dependent on the presence of peripheral B cells,^4^ and only CD19^+^HLA-DR^low^ PC, but not CD19^−^HLA-DR^low^ PC were detectable in the blood after vaccination, presumably as a result of their differential susceptibility to mobilization.^1^^,^^3^^,^^4^ Together, our data indicates that humoral SARS-CoV-2 immunity and memory is based on an array of different plasma cell types with different lifespan and stability^19^ potentially underlying subset-specific regulation. While the regulation of terminal differentiation events leading to the emergence of non-canonical CD19^−^CD45^+/−^ PC phenotypes remains to be revealed, our data show that significant numbers of non-canonical PC can emerge already after timely and quantitatively limited antigen exposure.

Taken together, our results support the early formation of SARS-CoV-2-specific CD19^+^ and CD19^−^ presumably long-lived plasma cells in the human BM after repeated mRNA vaccination against SARS-CoV-2, as an important prerequisite for durable serological immunity preventing severe courses of COVID-19.

## Contributors

ARS, LF, HH, LMD generated, analysed and verified the data, AN established protocols, SR and SH selected donors and provided samples. ARS and HEM conceptualised research. ARS and LF made the figures. ARS and HEM wrote the manuscript, and all other authors read, edited, and approved of the manuscript.

## Data sharing statement

Flow cytometry data of BM samples are available at FlowRepository.org. Accession number FR-FCM-Z68Q.

## Declaration of interests

HEM received public sector funding through the German Research Council (DFG) grants TRR130 TP24 and ME 3644/8-1; and through the Berlin Senate. SR received public sector funding through the German Federal Ministry of Education and Research (BMBF) grand SFB-1444 C01 Central Service Project–Cell and Tissue Harvesting: supply of functionally and immunologically characterised human samples for the CRC research projects. All other authors indicate that they have no competing interests.
