# Supplementary material for: SARS-CoV-2 specific plasma cells acquire long-lived phenotypes in human bone marrow
Source: eBioMedicine. 2023 Aug 8;95:104735. doi: 10.1016/j.ebiom.2023.104735 (PMC10432952; doi:10.1016/j.ebiom.2023.104735)
Supplement: Supplementary Figures S1–S3 [file mmc3.docx]

**SARS-CoV-2 specific plasma cells acquire the phenotype of long-lived plasma cells in the human bone marrow**

by Axel R. Schulz^1^, Leonard Fiebig^1^, Heike Hirseland^1^, Lisa-Marie Diekmann^1^, Simon Reinke^2^, Sebastian Hardt^3^, Antonia Niedobitek^1^, and Henrik E. Mei^1^

Supplementary methods information

3 figures, 2 tables


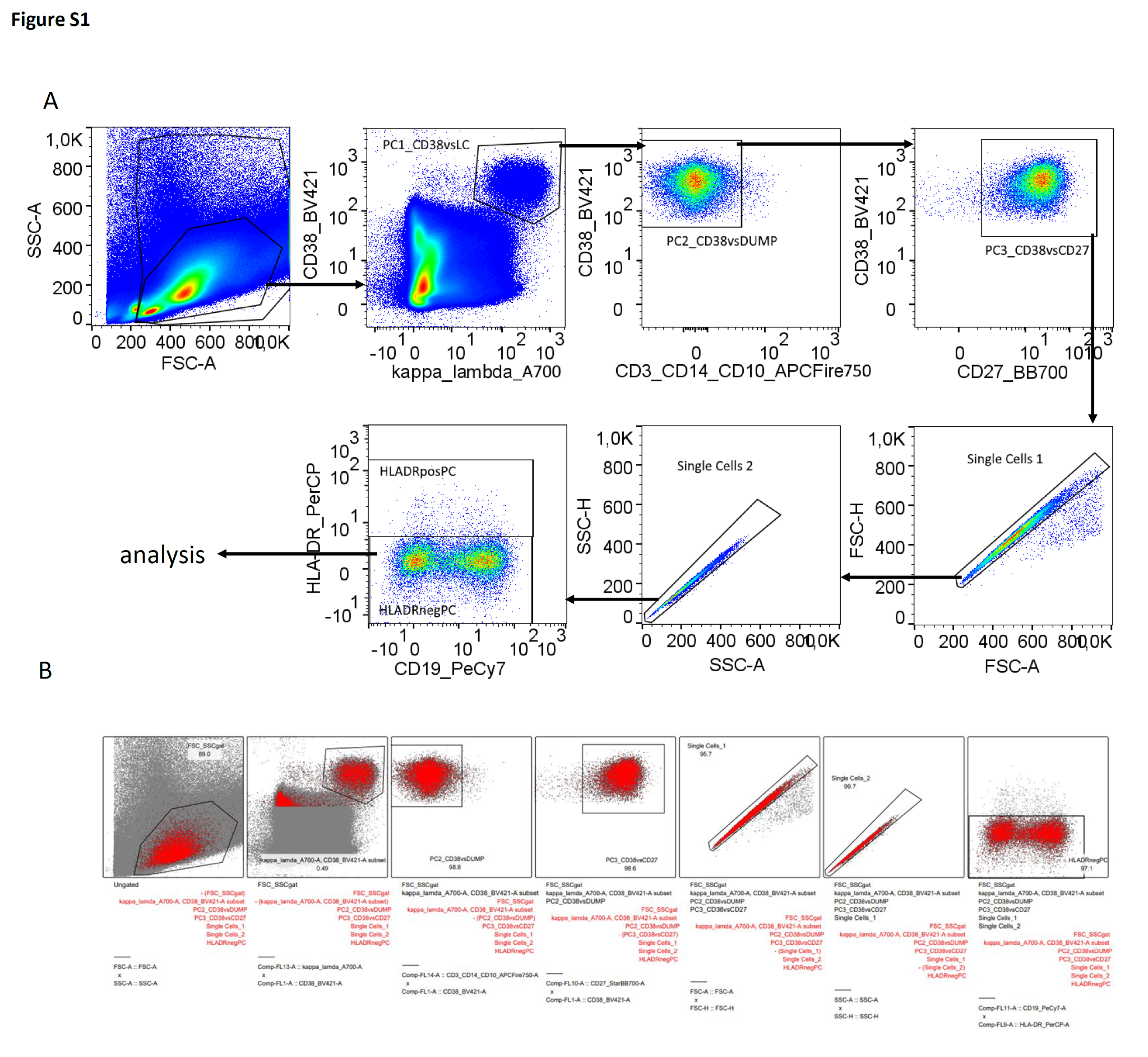


Figure S1. Flow cytometric gating strategy

(A) Total BM cells are shown in the first plot. The large gate was used to determine total numbers of acquired cells, the smaller gate was used to further gate on BMPC according to co-expression of high levels of CD38 and intracellular kappa/lambda antibody light chains in the subsequent plot. Additional gates were used to clean up the gated PC by excluding cells stained with antibodies against non-plasma cell lineages (CD3, CD14, CD10), by removing remaining CD27^low^CD38^++^ cells, cell aggregates in FSC-A vs FSC-H and SSC-A vs SSC-H plots, and finally excluding HLA-DR-expressing cells which may comprise precursors of bona fide PC, that is, plasmablasts (Mei et al., Blood, 2009). (B) Backgating of the same data shown in (A), verifying the above gating strategy. Data of one representative BM sample is shown (donor 2003).

**Figure S2**
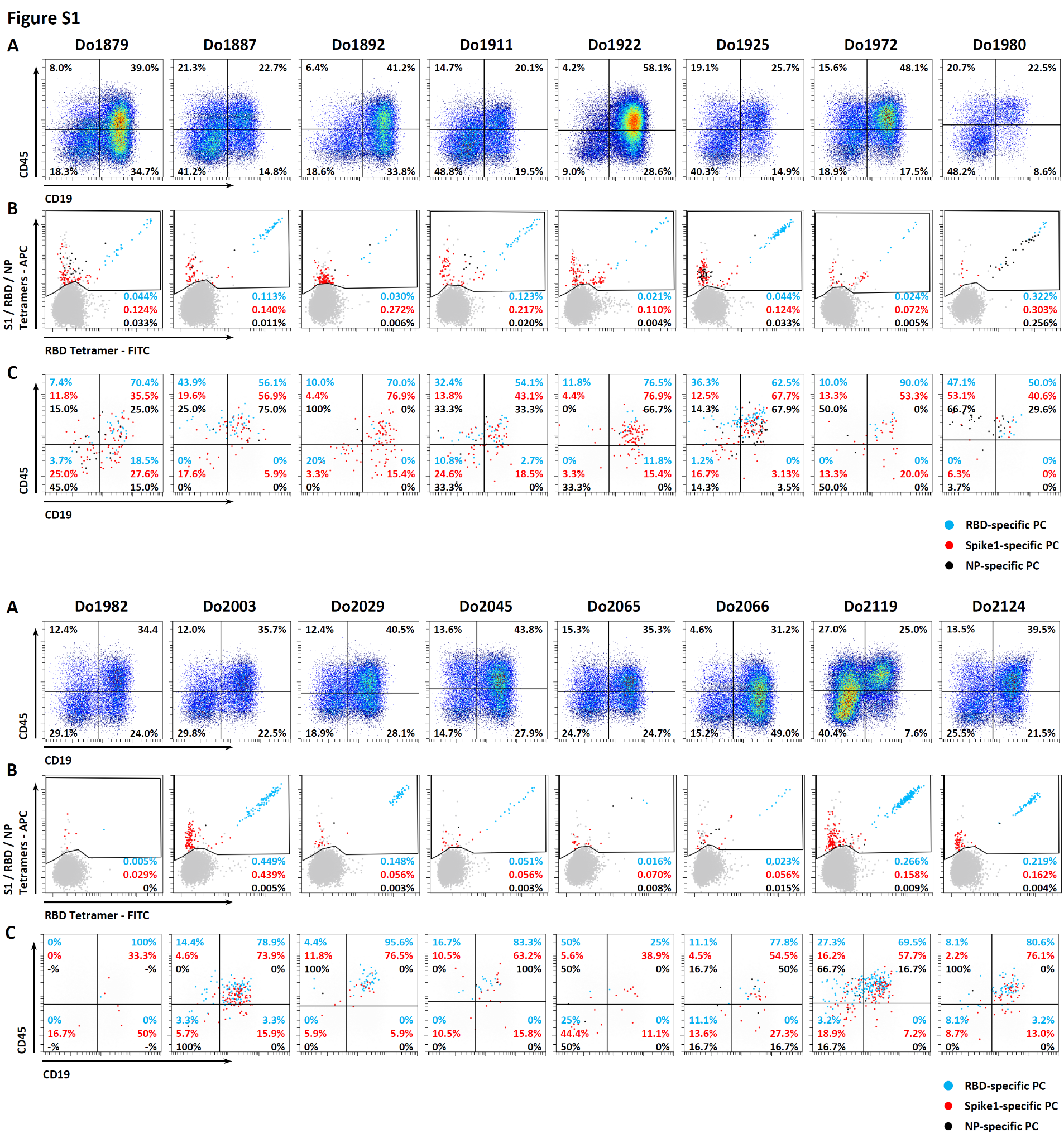


Figure S2. CD19/45 expression by total and antigen-specific BMPC, individual data.

BMPC were identified by flow cytometry as shown in Figures 1 and S1. (A) Expression of CD19 and CD45 by BMPC. (B) Overlay of PC specific for RBD, S1, or NP (gated as shown in Figure 1) in RBD-Tetramer-FITC vs S1/RBD/NP-Tetramers-APC plots. Numbers correspond to frequencies of total BMPC. (C) Overlay of RBD-, S1-, or NP-specific BMPC and their expression of CD19 and CD45. Numbers indicate frequencies of gated cells among RBD-, S1-, or NP-specific PC, respectively. Do, Donor

**Figure S3**


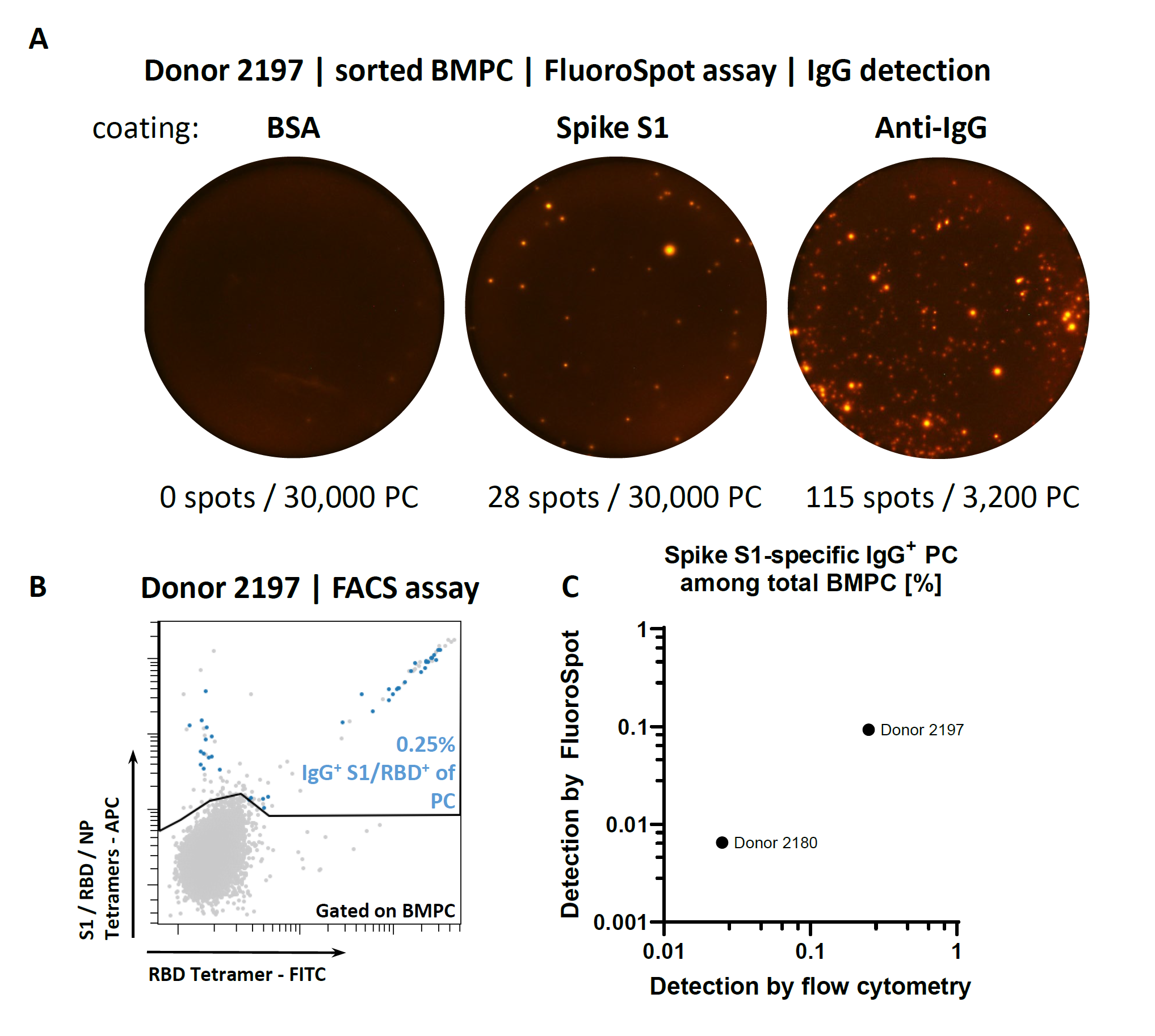


Figure S3. Confirmation of Spike S1 specificity by Fluorospot of BMPC

(A) PC were sorted from human BM cell suspension and indicated numbers of PC were seeded into wells of a FluoroSpot plate coated with BSA, Spike S1 antigen, or anti-human IgG. Detection by IgG-Cy3 revealed 0, 28, and 115 spots per well, respectively. The example shows PC of donor 2197. (B) Corresponding frequency of IgG^+^ S1/RBD-specific PC (blue layer) overlaid on total BMPC of donor 2197. (C) Comparison of the detection of Spike S1-specific IgG^+^ PC by flow cytometry versus FluoroSpot assay. Frequencies of donor 2180 and donor 2197 are shown
